# Assessment of Compliance With Infection Prevention Guidelines/Standard Precautions Among Healthcare Workers of Debark and Metema General Hospitals, Northwest Ethiopia

**DOI:** 10.1002/puh2.70417

**Published:** 2026-10-09

**Authors:** Mequanente Dagnaw, Meera Indracti, Dinesh Govindraj

**Affiliations:** ^1^ Department of Epidemiology and Biostatistics Institute of Public Health University of Gondar Gondar Ethiopia; ^2^ Department of Medical Biotechnology Institute of Biotechnology University of Gondar Gondar Ethiopia; ^3^ Department of Medical Biotechnology School of Allied and Healthcare Sciences Malla Reddy University Hyderabad Telangana India

**Keywords:** Ethiopia, healthcare workers, infection prevention, standard precautions, standard prevention

## Abstract

**Introduction:**

Healthcare‐associated infections remain a major public health problem, especially in low‐resource settings. Adherence to infection prevention (IP) guidelines reduces infection transmission and improves patient safety, yet poor compliance remains common in developing countries, including Ethiopia. Evidence from Debark and Metema General Hospitals is limited. Therefore, this study assessed compliance with IP guidelines and associated factors among healthcare workers (HCWs) in these hospitals in Northwest Ethiopia.

**Objective:**

To assess compliance with IP guidelines/standard precautions among HCWs at Debark and Metema General Hospitals, Northwest Ethiopia.

**Methods:**

An institution‐based cross‐sectional study was conducted among HCWs at Debark and Metema General Hospitals, Northwest Ethiopia. Data were collected using a structured pretested questionnaire and analyzed using SPSS version 26. Descriptive statistics and logistic regression analyses were assessing compliance with IP guidelines/standard precautions and associated factors. Variables with *p* < 0.25 in bivariable analysis were entered into the multivariable model. Associations were measured using crude odds ratio (COR) and adjusted odds ratio (AOR) with 95% confidence interval (CI), and *p* ≤ 0.05 will indicate statistical significance. Model fitness and multicollinearity tests were performed.

**Result:**

A total of 416 HCWs participated in the study, with a 100% response rate. The prevalence of good and poor compliance with IP guidelines/standard precautions was 42.06% and 57.94%, respectively. In multivariable logistic regression analysis, working in the maternal department, being aged 22–30 years, and easy access to IP materials were significantly associated with good compliance (*p* ≤ 0.05).

**Conclusion:**

Compliance with IP guidelines/standard precautions among HCWs was low in Debark and Metema General Hospitals. Working department, younger age, and availability of IP materials were significantly associated with good compliance. Improving access to IP materials and strengthening training and supervision are recommended to enhance compliance among HCWs.

## Graphical abstract

Healthcare‐associated infections remain a major challenge in low‐resource settings. This cross‐sectional study assessed compliance with infection prevention guidelines among 416 healthcare workers at Debark and Metema General Hospitals, Northwest Ethiopia. Overall, only 42.1% of healthcare workers demonstrated good compliance. Working in the maternal department, being aged 22–30 years, and having easy access to infection prevention materials were significantly associated with better compliance. Strengthening the availability of infection prevention materials, training, and supportive supervision is essential to improve compliance and enhance patient safety.

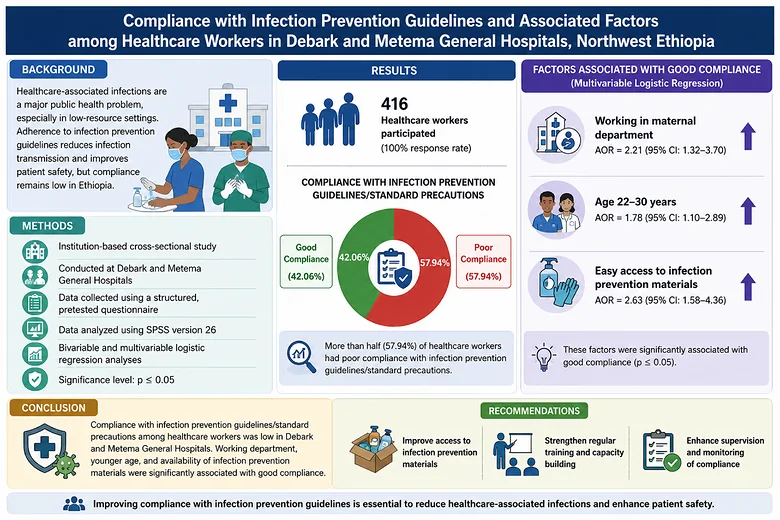

## 1 Introduction

Healthcare‐associated infections (HAIs) remain one of the most significant public health challenges worldwide, particularly in low‐ and middle‐income countries where healthcare systems face resource limitations and inadequate infection prevention (IP) infrastructure. Infection prevention and control (IPC) guidelines, especially standard precautions, are universally recommended measures designed to reduce the transmission of infectious agents in healthcare settings. These precautions include proper hand hygiene, use of personal protective equipment (PPE), safe handling of sharps, waste management, environmental sanitation, respiratory hygiene, and appropriate sterilization procedures. The World Health Organization (WHO) and the Centers for Disease Control and Prevention (CDC) emphasize that adherence to standard precautions is essential for protecting patients, healthcare workers (HCWs), and the community from HAIs and occupational exposure to blood‐borne pathogens [1, 2].

HCWs are continuously exposed to infectious materials through direct patient contact, contaminated instruments, and body fluids. Poor compliance with IP guidelines contributes to increased rates of HAIs, prolonged hospitalization, antimicrobial resistance, disability, and mortality. Globally, the WHO estimates that hundreds of millions of patients are affected annually by HAIs, with a disproportionately higher burden in developing countries due to overcrowding, shortage of PPE, inadequate water and sanitation facilities, and limited continuous professional training [1]. The COVID‐19 pandemic further highlighted the importance of strict adherence to IP practices in healthcare settings.

Several studies conducted in Ethiopia have shown that compliance with standard precautions among HCWs remains inadequate. A recent study conducted in Addis Ababa public hospitals reported that only 36.5% of HCWs adequately complied with IPC standard precautions [3]. Another study from Northeastern Ethiopia revealed that adherence to IP practices was significantly associated with availability of PPE, IP training, and institutional support systems [4]. Similarly, studies conducted in West Gojjam, Dessie, and Addis Ababa indicated that compliance levels ranged from 32.7% to 55.6%, suggesting persistent gaps in IP practices among healthcare professionals [5, 6, 7].

What is currently known is that HCWs who receive regular IPC training, have access to IP supplies, possess adequate knowledge, and work in facilities with active IP committees are more likely to comply with standard precautions [3, 4, 5]. Previous studies also identified factors, such as positive attitudes toward IP, supportive supervision, availability of written guidelines, and manageable workload, as important determinants of compliance [6, 7, 8, 9]. In addition, healthcare facilities with continuous monitoring and strong administrative commitment tend to achieve better adherence to IP practices.

Despite the existing evidence, important gaps remain insufficiently addressed. There is limited evidence regarding compliance with IP guidelines among HCWs in Debark and Metema General Hospitals, Northwest Ethiopia. These hospitals serve large and diverse populations, including mobile and border communities that may increase the risk of infectious disease transmission. Furthermore, little is known about the specific institutional and behavioral factors influencing compliance among HCWs in these settings. Most Ethiopian studies have focused on urban hospitals and cross‐sectional quantitative assessments, whereas evidence from remote and border areas remains scarce [10]. In addition, post‐COVID‐19 IP practices and challenges among HCWs in Northwest Ethiopia are not adequately documented.

Different efforts have been implemented globally and nationally to improve compliance with IP guidelines. WHO developed the IPC Core Components Guidelines and launched global hand hygiene campaigns to strengthen IPC practices in healthcare institutions [1]. Ethiopia's Ministry of Health has also introduced national IP and patient safety strategies, established IP committees in hospitals, and provided training programs for HCWs [11]. During and after the COVID‐19 pandemic, healthcare facilities received increased supplies of PPE, hand hygiene materials, and disinfectants to strengthen IP practices. Supportive supervision, routine monitoring, and HCW training have also been promoted in many healthcare institutions [3, 4, 11]. However, despite these interventions, compliance with standard precautions remains below recommended standards because of inadequate resources, inconsistent implementation of guidelines, poor supervision, and behavioral barriers among HCWs [4, 7, 12, 13]. Therefore, assessing compliance with IP guidelines among HCWs in Debark and Metema General Hospitals is crucial to identify existing gaps, determine associated factors, and provide evidence‐based recommendations that can improve healthcare quality, patient safety, and occupational health protection in Northwest Ethiopia.

## 2 Methods

### 2.1 Study Design and Period

An institution‐based cross‐sectional study was conducted to determine the assessment of compliance with IP guidelines/standard precautions among HCWs of Debark and Metema General Hospitals, Northwest Ethiopia, from November 2021 to June 2022.

### 2.2 Study Area

This study was conducted in general hospitals located in the Amhara region, Northwest Ethiopia. The most populated and second largest state of Ethiopia is the Amhara region. It has been divided into three cities and 10 zones. The regional health bureau is in the capital city of the Amhara region, namely, Bahir Dar. The total population of Amhara state, according to the health bureau report (2016), was 21.5 million, where 1.8 million was the rural population, and 19.7 million was the urban population.

A total of 4244 healthcare settings were found, consisting of 3336 health posts, 69 hospitals, and 839 health centers. Health posts in communities are the smallest primary health service centers, providing services to around 5000–8000 individuals [14].

Debark General Hospital is located in the northwest of Ethiopia, 890 km from Addis Ababa, the country's capital. Debark is a city in the North Gondar zone. There is only one general hospital in the North Gondar zone, which serves more than four districts. According to information collected from Debark General Hospital's nurse coordinator, there are 272 healthcare personnel working in different hospital departments, including emergency, ICU, recovery, operating room, inpatient, and outpatient. Debark citys’ total population was 227,526, consisting of 107,639 and 119,887 females and males, respectively, as reported by the Ethiopian agency of central statistics [15].

Metema General Hospital is located in the northwestern region of Ethiopia, 830 km from Addis Ababa, the country's capital. Metema is a city in the West Gondar zone. There is only one general hospital in the West Gondar zone, which serves more than four districts. According to information collected from the Metema General Hospital nurse's coordinator, 150 HCWs are working in several hospital departments, such as emergency, ICU, recovery, operating room, inpatient, and outpatient. The study area has a total population of 163,546, based on population projections for 2022. Of them, 87,192 (or 53.3% of the population) are men, and 76,354 (or 48.7%) are women [16].

### 2.3 Population

#### 2.3.1 Source Population

All healthcare workers are working at Debark and Metema General Hospitals.

#### 2.3.2 Study Population

During the study, all health workers working at Debark and Metema General Hospitals included physicians (general practitioners and surgeons), laboratory workers, anesthetists, nurses, radiology technologists, environmental health professionals, health officers, emergency surgeons, and midwives.

### 2.4 Inclusion and Exclusion Criteria

#### 2.4.1 Inclusion Criteria

All healthcare workers who are directly involved in daily client care (e.g., HCWs working in medical, surgical, obstetrics and gynecology, pediatrics, outpatient departments [OPDs], maternal and child health (expanded immunization, family planning, and antenatal care), emergency, laboratory, and radiology departments).

#### 2.4.2 Exclusion Criteria

HCWs are less likely to have physical contact with patients, such as health service administration staff members, and HCWs who have worked for less than 6 months.

### 2.5 Study Variables

#### 2.5.1 Dependent Variables

Dependent variables are complied with standard precautions.

#### 2.5.2 Independent Variables

Respondents’ age, gender, professional category, present working department, total work experience, availability of IP equipment, and ease of access to IP equipment.

### 2.6 Operational Definitions

#### 2.6.1 Good Compliance With Standard Precautions/IP Practices

HCWs who score ≥80% of the total standard precaution/IP practice items shall be classified as having good compliance. This threshold was adapted from the Bloom‐derived scoring approach and is consistent with previous studies assessing HCWs’ compliance with standard IP precautions, where scores of 80%–100% indicate good practice/compliance [17, 18].

#### 2.6.2 Poor Compliance With Standard Precautions/IP Practices

HCWs who score <80% of the total standard precaution/IP practice items shall be classified as having poor compliance. For dichotomized analysis, all scores below the 80% threshold are categorized as poor compliance, consistent with the adapted Bloom‐based classification used in previous IPC compliance studies [17, 18].

### 2.7 Sample Size Determination

As published research conducted on the practice of standard guidelines among health professionals in Hawassa in 2020 shows that 56.5% of HCWs have compliance with standard precautions, we have used *p* = 56.5% [19]. Then we used the single population proportion formula as follows:

*p* = 56.5%, and *p* + *q* = 1 (this means *p* = 0.565 and *q* = 0.435)

CI = 95% confidence interval, Za/2 = 1.96, *d* = ±5%

$$n=\frac{{\left(\mathrm{Za}/2\right)}^{2}\left(pq\right)}{d^{2}}$$

$$n=\frac{{\left(1.96\right)}^{2}\left(0.565\right)\left(0.435\right)}{{\left(0.05\right)}^{2}}=378$$

where ni What is the initial sample size?

After this, we considered adding 10% nonrespondents (contingency), which is equal to 38. By doing this, the total sample size was **416** healthcare workers.

### 2.8 Sampling Procedure

The study was conducted among HCWs employed at Debark General Hospital and Metema General Hospital in Northwest Ethiopia. Initially, the total number of HCWs in both hospitals was obtained from the human resource departments of each hospital. The calculated sample size was then proportionally allocated to each hospital on the basis of the number of HCWs working in the respective hospitals to ensure proportional representation. The proportional allocation for each hospital was determined using the following formula:

$$\;n_{i}=\frac{N_{i}}{N}\times n$$

where $n_{i}$ is the sample size allocated to each hospital. $N_{i}$ is the total number of HCWs in each hospital. N is the total number of HCWs in both hospitals. n is the total final sample size.

After proportional allocation was completed, a sampling frame containing a list of all eligible HCWs was obtained from the human resource offices of Debark and Metema General Hospitals. HCWs who fulfilled the inclusion criteria were included in the sampling frame. Finally, study participants were selected from each hospital using a simple random sampling technique through the lottery method. Each eligible HCW was assigned a unique identification number, and the required number of participants was selected randomly until the allocated sample size for each hospital was achieved. This method ensured that every HCW had an equal chance of being included in the study.

### 2.9 Data Collection Tool and Procedure

Quantitative data were collected using a standardized semi‐structured self‐administered questionnaire prepared in English. The questionnaire was adapted from the validated Compliance with Standard Precautions Scale (CSPS), a widely used instrument for assessing HCWs’ adherence to IP and standard precaution practices [20, 21]. The tool consisted of items assessing respondents’ sociodemographic characteristics, compliance with recommended IP precautions, and the availability and accessibility of IP materials and equipment. Previous studies have demonstrated that the CSPS has good psychometric properties, including acceptable validity and reliability, with Cronbach's alpha values greater than 0.80 [21, 22]. In the current study, compliance scores were calculated by summing responses to individual compliance items, and the total score was treated as a continuous outcome variable during regression analysis after assessment of normality assumptions and application of appropriate transformation where necessary [3, 23]. The data were collected for 30 days in a row. The sociodemographic and individual factors questionnaires were adapted from the previous study. The data collector was delivering questionnaires to research participants and informed them to read the instructions and consent form carefully.

### 2.10 Data Quality Control

The questionnaire was developed in English. Before data collection, 5% of the total study‐appropriate participants other than the selected hospital were pretested, and they shared the same features as the health institution that was Wogera Primary Hospital. The lead investigator provided intensive 1‐day training for data collectors and supervisors on the objectives, tools, and procedures of data collection, as well as how to safeguard the anonymity of study subjects. Three diploma nurses were assigned for data collecting, one BSc nurse was assigned as an investigator, and one BSc nurse was assigned to supervise the entire data gathering. During the data‐collecting period, supervisors were responsible for ensuring that the questionnaire was correctly filled out and for closely monitoring the data.

### 2.11 Data Processing and Analysis

For inferential and descriptive analysis, the data were put into the 3.1 version of EPI and subsequently STATA (version 18). For investigating the impact of independent variables on dependent variables, binary logistic regression was taken into account. The *p* value less than 0.25 of the bivariate analysis was subjected to multivariable regression analysis for estimation of the predictor variables for the level of compliance with IP guidelines/standard precautions. Before further analyses were carried out using multivariable analysis at a significance level of *p* value ≤ 0.05, multicollinearity was checked among selected independent variables using the variance inflation factor, and none was found. Before conducting the final analysis, an exploratory analysis was performed to check for missing values, outliers, and assumptions. Variables having a *p* value less than 0.05 were considered statistically significant in the final regression, and the fitness of the model was evaluated at *p *> 0.05 by Hosmer–Lemeshow.

Odds ratios with 95% confidence intervals were used to assess the direction and degree of statistical associations.

## 3 Results

### 3.1 Sociodemographic Characteristics of Respondents

A self‐administered questionnaire was distributed to 416 healthcare practitioners. Of them, all 416 HCWs completed and returned the questionnaire, yielding a response rate of 100% with the substitutions of nonrespondent participants. The respondents’ ages ranged from 22 to 30 years, with a mean of 30.30 years (SD‐+ = 3.655 years). The majority of HCWs, 210 (50.48%), are between the ages of 22 and 30. The majority of the responders, 218 (52.40%), were female, whereas 198 (47.60%) were male. Out of 416 study participants, 250 (60.09%) were unmarried, and 39.40% were married (Table 1).

**TABLE 1:** Sociodemographic characteristics of the assessment of compliance with infection prevention guidelines/standard precautions among healthcare workers of Debark and Metema General Hospitals, Northwest Ethiopia, 2022 (*n* = 416).

Variable	Category	Frequency	Percent
Age of participants	22–30 years	210	50.48
	31–40 years	206	49.52
Sex	Male	198	47.60
	Female	218	52.40
Marital status	Married	166	39.90
	Unmarried	250	60.09
Working department	Inpatient	204	49.04
	Maternal	130	31.25
	OPD	82	19.71
Profession	Doctor/physician	43	10.34
	Laboratory technician	55	13.22
	Health officer	29	6.97
	Emergency surgeon	18	4.33
	Anesthetics	10	2.40
	Nurse	201	48.32
	Midwives	50	12.02
	Environmental worker	3	0.72
	Radiology	7	1.68
Work experiences	<5 years	205	49.28
	≥5 years	211	50.72

Abbreviation: OPD, outpatient department.

### 3.2 Compliance With IP and Standard Precautions

Out of 198 male participants, 98 (23.56%) demonstrated poor compliance with IP and standard precautions. Almost half of the 118 female participants (28.37%) exhibited good compliance with IP and standard procedures (Table 2).

**TABLE 2:** Distribution of sociodemographic‐related variables for compliance with infection prevention of the assessment of compliance with infection prevention guidelines/standard precautions among healthcare workers of Debark and Metema General Hospitals, Northwest Ethiopia, 2022 (*n* = 416).

Variables	Category	Compliance with IP		Total
		Poor compliances	Good compliances	
Age of respondents	22–30 years	117 (28.13%)	99 (23.80%)	216
	31–40 years	110 (26.44%)	90 (21.63%)	200
Sex	Male	98 (23.56%)	100 (24.04%)	198
	Female	100 (24.04%)	118 (28.37%)	218
Marital statues	Married	94 (22.60%)	113 (27.16%)	217
	Unmarried	100 (24.04%)	99 (23.80%)	199
Work department	Inpatient	58 (13.94%)	46 (11.06%)	114
	Maternal	47 (11.30%)	45 (10.82%)	92
	Out Patients	56 (13.46%)	44 (10.58%)	100
Experiences	<5 years	102 (24.52%)	104 (25%)	206
	≥5 years	112 (65.5%)	98 (23.56%)	210

### 3.3 Perceived Responses of Accessibility and Availability of IP Materials

Among 416 study participants, 29.1% reported that IP resources were available, whereas 36.45%, 15.89%, 14.95%, and 2.80% reported that IP materials were occasionally available, rarely available, generally available, or not available, respectively. Regarding the easy accessibility of IP resources, 67% of respondents responded that IP materials were easily accessible and available, whereas 33% responded that IP materials are not easily accessible and available (Figure 1).

**FIGURE 1:**
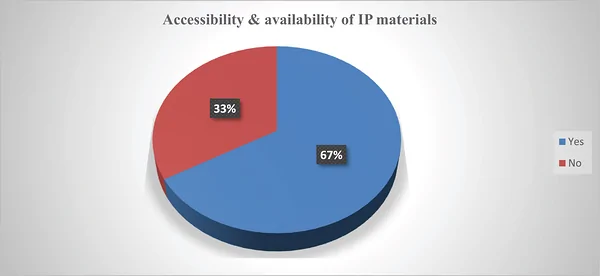
Perceived responses of accessibility and availability of infection prevention materials of the assessment of compliance with infection prevention guidelines/standard precautions among healthcare workers of Debark and Metema General Hospitals, Northwest Ethiopia, 2022 (*n* = 416).

### 3.4 Compliance With IP and Standard Precautions Among HCWs

The majority of healthcare personnel, 138 (33.17%), washed their hands most of the time before completing a procedure. The majority of healthcare workers, 150 (36.06%), used eye covers/goggles before performing procedures (Table 3).

**TABLE 3:** Compliance with standard guidelines and infection prevention of the assessment of compliance with infection prevention guidelines/standard precautions among healthcare workers of Debark and Metema General Hospitals, Northwest Ethiopia, 2022 (*n* = 416).

Variable	Category	Frequency	Percentage
Hands wash	Not know	84	21.19
	Rarely	94	22.60
	Sometimes	100	24.04
	Most of the time	138	33.17
Hand washing after the procedure	I do not wash my hands	80	19.23
	Rarely	98	23.56
	Sometimes	108	25.96
	Most of the time	130	31.25
Wear a gown during the procedure	I do not wear	88	21.15
	Rarely	90	21.63
	Sometimes	118	28.37
	Most of the time	120	28.85
Masks are used to perform a procedure	I do not wear	70	16.83
	Rarely	108	25.96
	Sometimes	116	27.88
	Most of the time	138	33.17
Use eye cover/goggles	I do not use	12	2.88
	Rarely	104	25
	Sometimes	150	36.06
	Most of the time	150	36.06
Wear glove	I do not wear	22	5.29
	Rarely	26	6.25
	Sometimes	88	21.15
	Most of the time	280	67.31
Reuse needles or syringes	Always	14	3.37
	Oftentimes	12	2.88
	Sometimes	88	21.15
	Rarely	52	12.5
	I never reuse needles	250	60.10
Recap needles	Always	10	2.40
	oftentimes	16	3.85
	Sometimes	61	14.66
	Rarely	129	31.00
	I never recap needles	200	48.08
Dispose of used needles	I do not dispose	4	0.96
	Rarely	20	4.81
	Sometimes	114	34.62
	Most of the time	126	30.29
	All the time	152	36.54
Non‐sharp wastes disposed	Not disposed of properly	80	19.23
	Rarely	60	14.42
	Sometimes	66	15.87
	Most of the time	154	37.02
	all the time	56	13.46

### 3.5 Compliance With IP and Standard Precautions

The compliance of health workers with IP and standard precautions at Debark and Metema General Hospitals was tested by calculating the mean of 10 factors. The incidence of poor compliance among healthcare personnel was 57.94%, whereas 42.06% had good compliance (Figure 2).

**FIGURE 2:**
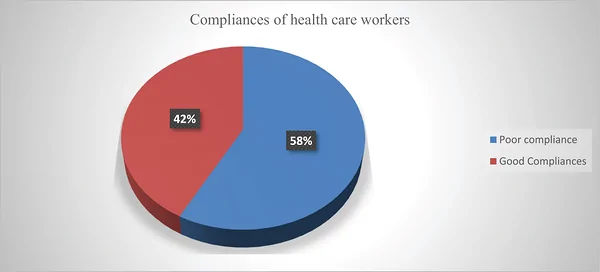
Compliance of healthcare workers. The assessment of compliance with infection prevention guidelines/standard precautions among healthcare workers of Debark and Metema General Hospitals, Northwest Ethiopia, 2022 (*n* = 416).

### 3.6 Factors Associated With Compliance With IP and Standard Precautions

First, a bivariate analysis was carried out to determine the factors that influence IP and standard precautions compliance. The multivariable analysis included sex, age, access to infection control tools, working department, and HCW experience (*p* < 0.25). The multivariable model found that the working department, age of respondents, and easy access to IP materials were significant predictors of compliance with IP standards among HCWs at Debark and Metema General Hospitals (*p* ≤ 0.05).

Given that all other factors in the model were fixed, healthcare professionals in the maternal ward had 2.98 times greater odds of complying with IP and standard precautions than those in the OPD (AOR = 2.98, 95% CI = 1.13, 7.83). Given all variables’ constant, the probabilities of prevention of infections and standard guidelines compliance in health professionals.

Aged 22–30 years were 2.46 times greater than among healthcare workers aged 31–40 years (AOR = 2.46, 95% CI = 1.13, 5.32). The probability of having compliance with IP and standard precautions was decreased by 74% for those healthcare professionals assuming not easily accessible IP materials than those assuming easily accessible IP materials (AOR = 0.26, 95% CI = 0.13,0.54), given (Table 4).

**TABLE 4:** Results of bivariable and multivariable Binary logistic regression of the assessment of compliance with infection prevention guidelines/standard precautions among healthcare workers of Debark and Metema General Hospitals, Northwest Ethiopia, 2022 (*n* = 416).

Variables	Category	COR with 95% CI	*p* value	AOR with 95% CI	*p* value
Sex	Male	1.885 (1.018, 3.488)	0.044	0.675 (0.341, 1.336)	0.259
	Female	1		1	
**Easy IP material accessibility**	Not easily accessible	2.528 (1.371, 4.660)	0.003	0.263 (0.129, 0.536)	**0.000**
	Easily accessible	1		1	
Work experiences	<5 years	1.979 (1.141, 3.433)	0.015	1.029 (0.464, 2.283)	0.944
	>5 years	1		1	
**Department**	Inpatient	1.525 (0.827, 2.812)	0.176	1.511 (0.739, 3.091)	0.258
	Maternal	2.163 (0.950, 4.929)	0.66	2.979 (1.134, 7.825)	**0.027** ^*^
	Outpatient	1		1	
**Age**	22–30 years	2.351 (1.339, 4.127)	0.003	2.456 (1.133, 5.324)	**0.023** ^*^
	31–40 years	1		1	

Bold value shows signifacnt variable *p*‐value.

Abbreviations: AOR, adjusted odds ratio; CI, confidence interval; COR, crude odds ratio.

* Statistically significant at *p* value ≤ 0.05.

## 4 Discussion

The purpose of this study was to analyze the self‐reported level of compliance about infection control and standard precautions, as well as the associated factors, among healthcare personnel at Metema and Debark General Hospitals in Northwest Ethiopia. The findings of the current study reveal that the prevalence of compliance with IP and standard precautions among HCWs was 42.06%, which is much lower than that in the study conducted in China (48.29%) of participants who had good compliance with IP and standard precautions [24]. This variance could be explained by differences in research area, hospital policies, workload, availability of resources, and training opportunities, lack of well‐trained HCWs, inadequate IPC policies, limited resources, and variable compliance among HCWs, study population, and socioeconomic status, as well as organizational and administrative discrepancies [25]. In China, norms and regulations addressing the proper implementation of standard precautions, the availability of infection control items, and the regular monitoring of health workers were substantially different from the study context [26].

It has been found in this study that the percentage of conformation to IP and standard precautions was lower than the study conducted in Egypt (57.5%) [27]. Methodological variations could explain this variation. In Egypt, the study subjects were surgeons, whereas in our study location, the study subjects were all healthcare staff. This educational variation may result in a gap in compliance levels [28].

Compliance with IP and routine prescriptions was lower in this study than in the Zambian study (66%) of participants [29]. These differences could be explained by variances in organizational structures between countries. Resource‐constrained countries had low feasibility of IP materials.

Our investigation found that 57.94% of study participants had low compliance with IP, which was lower than a study conducted in Jimma (69.7% of respondents had inadequate compliance with SPs) [30]. One possible explanation for these disparities is that Jimma's study only included nurses, but our current analysis included emergency surgeons and doctors with greater education levels than nurses. The cumulative sum of the results may vary. The level of compliance among HCWs, according to the study conducted in Mekele, Ethiopia, was 42.9% of the participants who had adequate IP measures [31]. The likely reason for this resemblance could be the similarity of the research subject's profession and the similar hospital standards.

This study revealed compliance with IP, which was lower than the study conducted in Gondar, 65% [32] and 56.5% [19] of healthcare had complained about IP safety [19]. One possible explanation for this variation is research conducted in Gondar on comprehensive and specialty hospitals that found significant disparities in access to infection control tools and regular monitoring of healthcare staff. In Gondar hospitals, advanced procedures requiring tight IP were implemented, allowing the trends to be sustained. Furthermore, at Gondar referral hospitals, the staff has a greater level of knowledge, which aids in the correct maintenance of intellectual property.

The current study found that age, ease of access to IP materials, and working department were important predictors of compliance with IP and standard precautions among HCWs at Debark and Metema General Hospitals.

In this study, age was identified as one of the characteristics related to IP compliance. Given that all other variables remained constant, healthcare professionals aged 22–30 were 2.46 times more likely to comply with IP and standard precautions than HCWs aged 31–40 years [AOR = 2.456 (1.133, 5.324)]. This outcome was similar to the studies undertaken in Tanzania [33] and Germany [21]. This may be because younger workers are more recently trained, more updated on IP guidelines, and more likely to follow standard precautions strictly.

The present study revealed that healthcare personnel who perceived IP materials as difficult to access were 74% less likely to comply with IP practices compared to those who reported easy accessibility. This finding is consistent with the other study conducted in Ethiopia [34], which showed that the availability of IP supplies significantly improved compliance among HCWs. Similarly, a study in China [35] reported that inadequate access to PPE and hand hygiene materials reduced adherence to standard precautions. Another study in Uganda [36] also found that shortages of IP materials were associated with poor compliance practices among HCWs.

The possible reason for this association may be that inadequate access to IP materials, such as gloves, masks, hand sanitizers, and disinfectants, limits HCWs’ ability to consistently practice standard precautions. In addition, frequent shortages may reduce motivation and increase unsafe practices during routine patient care.

The present study found that working department was a significant predictor of IP compliance, with HCWs in the maternal ward showing better compliance than those working in the OPD. This finding is consistent with the study conducted in Ethiopia, which reported higher IP practice among HCWs in maternity and inpatient units compared to OPD staff. Similarly, study found that HCWs in high‐risk clinical areas demonstrated better adherence to standard precautions than those in outpatient settings [37]. A possible reason for this difference may be that maternal wards frequently handle labor, delivery, and surgical procedures, where the risk of exposure to blood and body fluids is higher. This may increase HCWs’ awareness and adherence to IP measures. In addition, surgeries and procedures in maternal wards are often planned and performed during the day, allowing better supervision, availability of supplies, teamwork, and adherence to protocols compared to the busy and overcrowded OPDs.

The present study found that regular in‐service training on IPC practices significantly improved HCWs’ compliance with IP measures. This finding is consistent with studies conducted globally, in Africa, and in Ethiopia.

Globally in China, it was reported that HCWs who received regular IPC training demonstrated better adherence to standard precautions than those without training [38]. Similarly, in the United States, it was found that continuous education and training programs positively influenced compliance with IP guidelines [39].

In Africa, a study done in Uganda showed that HCWs who attended IPC training were more likely to practice proper IP measures [36]. Likewise, in Nigeria reported that regular training improved knowledge and compliance with standard precautions among healthcare professionals [40].

A study done in Ethiopia found that HCWs who received IPC‐related training had significantly higher compliance with IP practices compared to those without training and also reported that in‐service training was strongly associated with improved IP practice among HCWs [34].

The possible reason may be that regular training increases HCWs’ knowledge, awareness, and practical skills regarding IP measures. Training also helps staff stay updated with current guidelines, improves attitudes toward patient safety, and reinforces correct use of PPE and hand hygiene practices. In addition, repeated training may strengthen confidence and encourage consistent adherence to standard precautions in daily clinical practice.

## 5 Conclusion

In this study, healthcare personnel had a low rate of IP and standard procurement compliance. Healthcare personnel who have easy access to IP material, work in the maternal department, and are between the ages of 22 and 30 are substantially more likely to comply with IP and standard procedures. Hospitals should enhance training and ensure a consistent supply of PPE and IP materials to improve compliance. These studies highlight the need for comprehensive, evidence‐based policies that support effective infection control practices across all levels of healthcare delivery. Education and training, availability of resources and equipment, workload and staffing infrastructure and environment, monitoring and feedback, policy and guidelines, and motivation and supervision are factors that need intervention for healthcare facilities that can significantly improve IP compliance and reduce HAIs.

## 6 Strengths and Limitations of the Study

A limitation of this study is that compliance with IP and standard precautions was assessed using self‐administered questionnaires, which may be subject to self‐report and social desirability bias. Healthcare professionals might have overreported desirable IP practices because noncompliance can carry professional and social stigma. Consequently, the reported level of compliance may have been overestimated compared with actual routine practice. To reduce this bias, anonymity and confidentiality were assured, participants were informed that the data would be used solely for research purposes, and questionnaires were completed without the involvement of supervisors or hospital administrators. Although observational cross‐validation or direct practice audits could have strengthened the validity of the findings, these methods were not feasible due to limited resources, time constraints, and the busy clinical environment. Future studies are, therefore, recommended to incorporate observational methods or mixed‐method approaches to validate self‐reported compliance data and minimize social desirability bias.

## Author Contributions

Mequanente Dagnaw initiated the idea of research, conceptualizing, developing a proposal, doing analysis, and writing up the manuscript. Mequanente Dagnaw, Dinesh Govindraj., and Meera Indracti doing analysis, reviewing the manuscript, and correcting it. Finally, all the authors have approved the manuscript, by preparing it for submission.

## Funding

The authors have nothing to report.

## Ethical Statement

Ethical clearance was obtained from Internal Review Board (IRB) of GT Technology College with the reference no. Ref No/GTTC 22/03/2022 and then submitted to Debark and Metema General Hospitals. Before collecting data from healthcare professionals, the purpose of the study was explained to the participants, and data would be collected after ensuring their willingness. All ethical principles are performed in accordance with the Declaration of Helsinki.

## Consent

Informed written consent was obtained from the study participants before the conduct of the study.

## Conflicts of Interest

The authors declare no conflicts of interest.

## Data Availability

The corresponding author will have the right access to the data upon request.
